# Correlative Fluorescence and Scanning Electron Microscopy of Labelled Core Fucosylated Glycans Using Cryosections Mounted on Carbon-Patterned Glass Slides

**DOI:** 10.1371/journal.pone.0145034

**Published:** 2015-12-21

**Authors:** Marie Vancová, Jana Nebesářová

**Affiliations:** 1 Institute of Parasitology, Biology Centre of the Czech Academy of Sciences, v.v.i, České Budějovice, Czech Republic; 2 Faculty of Science, University of South Bohemia, České Budějovice, Czech Republic; 3 Faculty of Science, Charles University in Prague, Prague, Czech Republic; University of Maryland, College Park, UNITED STATES

## Abstract

The aim of the study is co-localization of N-glycans with fucose attached to N-acetylglucosamine in α1,3 linkage, that belong to immunogenic carbohydrate epitopes in humans, and N-glycans with α1,6-core fucose typical for mammalian type of N-linked glycosylation. Both glycan epitopes were labelled in cryosections of salivary glands isolated from the tick *Ixodes ricinus*. Salivary glands secrete during feeding many bioactive molecules and influence both successful feeding and transmission of tick-borne pathogens. For accurate and reliable localization of labelled glycans in both fluorescence and scanning electron microscopes, we used carbon imprints of finder or indexed EM grids on glass slides. We discuss if the topographical images can provide information about labelled structures, the working setting of the field-emission scanning electron microscope and the influence of the detector selection (a below-the-lens Autrata improved YAG detector of back-scattered electrons; in-lens and conventional Everhart-Thornley detectors of secondary electrons) on the imaging of gold nanoparticles, quantum dots and osmium-stained membranes.

## Introduction

*N*-Glycosylation belongs to a major modification of proteins, however, glycan structures differ among organisms. For example, in insects and plants, fucose can be linked to the proximal *N*-acetylglucosamine of a common *N*-glycan pentasaccharide core by α1,3-linkage, that is absent on mammalian carbohydrate structures. In contrast, α1,6-linkage of fucose to the N-glycan core is widespread in both invertebrate and vertebrate [1, 2]. In ticks, the presence of host glycosylated molecules complicates the research on glycan molecules; however, high structural similarity to glycans of insects can be assumed [3–5]. The core α1,3-fucose can induce production of specific IgE antibodies associated with IgE-mediated allergic immune responses in mammals to various invertebrate organisms, e.g. schistosomes or venoms of hymenoptera species, but not to ticks [6, 7]. Therefore, we focused on identification of glycans carrying α1,3-core fucose in salivary glands of ticks *I*. *ricinus* to find out the reason. Salivary glands of ticks are complex organs composed from three morphologically distinct types of acini. The acini of type I are involved in osmoregulation and absorption of water during off-host period. The most abundant acinus type III contains both granular cells and cells forming the basal labyrinth that have a water transport function [8]. The cells of the type II acini are formed only by granular cells that increase their size and number of secretory granules during tick feeding. These cells secrete various pharmacologically active biomolecules that influence haemostatic, inflammatory and immuno responses of hosts during the tick engorgement.

For visualization of core fucosylation of N-glycans on thawed cryosections, we used the correlative fluorescent and scanning electron microscopy (SEM) approach as an alternative to widely used fluorescent and transmission electron microscopy (TEM) combination. Both SEM and TEM produce images of biological structures at high resolution, but based on different origin of contrast. SEM can use different signals for imaging of biological structures, e.g. topographical information can be revealed by the detection of secondary electrons (SE), and imaging in backscattered electrons (BSE) is suitable for the detection and differentiation of metal nanoparticles (NPs) embedded in a cell matrix [9–11]. SEM due to large chamber may accommodate several other detectors/instruments with the aim to gain information about complex objects or rare structures at different magnifications (e.g. widefield epifluorescence microscope/objective lens inside the SEM chamber) or offer to study the three dimensional organization of structures (serial block face imaging, focused ion beam milling) [12, 13].

Next, samples can be imaged in SEM at different accelerating voltage. A higher accelerating voltage enlarge the interaction volume from which the electrons are scattered [14–16]. In ultrathin sections, SEM working in the transmission mode at higher accelerating voltage provide a transmission image of cell structures similarly to TEM [16, 17]. At accelerating voltage below 5 kV, SEM provides detailed information on the surface morphology with minimal charging and radiation damage of sensitive (either dried or hydrated) biological samples.

The significant advantage of using SEM for correlative imaging is that an object in large quantity or even whole mounts can be observed.

## Materials and Methods

### Preparation of cryosections and carbon-patterned coverslips

Salivary glands were isolated from females *Ixodes ricinus* partially fed for 6 days on clean guinea pigs. All animals used in this study was carried out in strict accordance with the Animal Protection Law of the Czech Republic No. 246/1992. The protocol was approved by the Committee on the Ethics of Animal Experiments of the Institute of Parasitology, Biology Centre of the Academy of Sciences of the Czech Republic, ethics approval No. 75/2013.

Samples were fixed in 4% formaldehyde/0.1% glutaraldehyde in 0.1 M phosphate buffer (PB) for 1 hr at room temperature. After washing in 0.01 M glycine in PB, the samples were immersed in 2.3 M sucrose for 3 days at 4°C and then frozen by plunging into liquid nitrogen. Cryosections were cut using a cryoultramicrotome Leica EM FCS (Leica Microsystems) and removed from the cryochamber Leica UCT using a drop of 2.3 M sucrose/2% methyl cellulose (1:1) [18]. The sections were thawed and placed on the coverslips with the carbon imprints of index EM grids (SPI) or finder grids (Leica Microsystems). The imprints were produced on the surface of glass coverslips washed carefully with ethanol. The grids were fastened with double-sided adhesive carbon tape and coated either with a thick carbon layer using the evaporator JEOL JEE 4C or gold layer by means of the sputter coater (Baltec SCD 0–50). The copper grids were removed, and the slides were washed in ethanol and dried at 60°C for 48 h. Schematic diagram is shown in S1 Fig.

### Fluorescent staining of glycans

Cryosections on coverslips were washed in buffer and blocked with a solution containing 3% of BSA/0.2% glycine in 0.1 M HEPES for 2 hours at RT, and incubated with biotinylated *Lens culinaris* agglutinin (LCA; Vector Laboratories) at a working concentration of 25 μg/ml. Rabbit polyclonal anti-α1,3-linked core-fucose serum was applied at a concentration of 3 μg/mL (Agrisera). All probes were diluted in 0.5% BSA and incubated with the sections for 2 hours. After washing with 0.5% BSA in HEPES, immunostaining was detected with Extravidin—fluorescein isothiocyanate (FITC) (Sigma-Aldrich, 15 μg/ml) and anti-rabbit Fab´IgG conjugated to Q dot 605 (QDs, Molecular Probes, 20 nM) for 1 h. Negative control experiments were performed by omitting the glycan-binding probes and specificity of glycan-binding probes was tested as described in the part supporting information (S1 Protocol, S2 Fig and S1 Table). The sections were washed in HEPES and stained in DAPI (Sigma-Aldrich, 0.01 μg/ml, 5 min). After rinsing, the coverslips with section side down were placed on buffer. Fluorescence was examined using an Olympus BX 51 fluorescent microscope. Immediately after the observations, the coverslips were placed section side down on buffer.

### Gold labelling and SEM sample preparation

**Fig 1:**
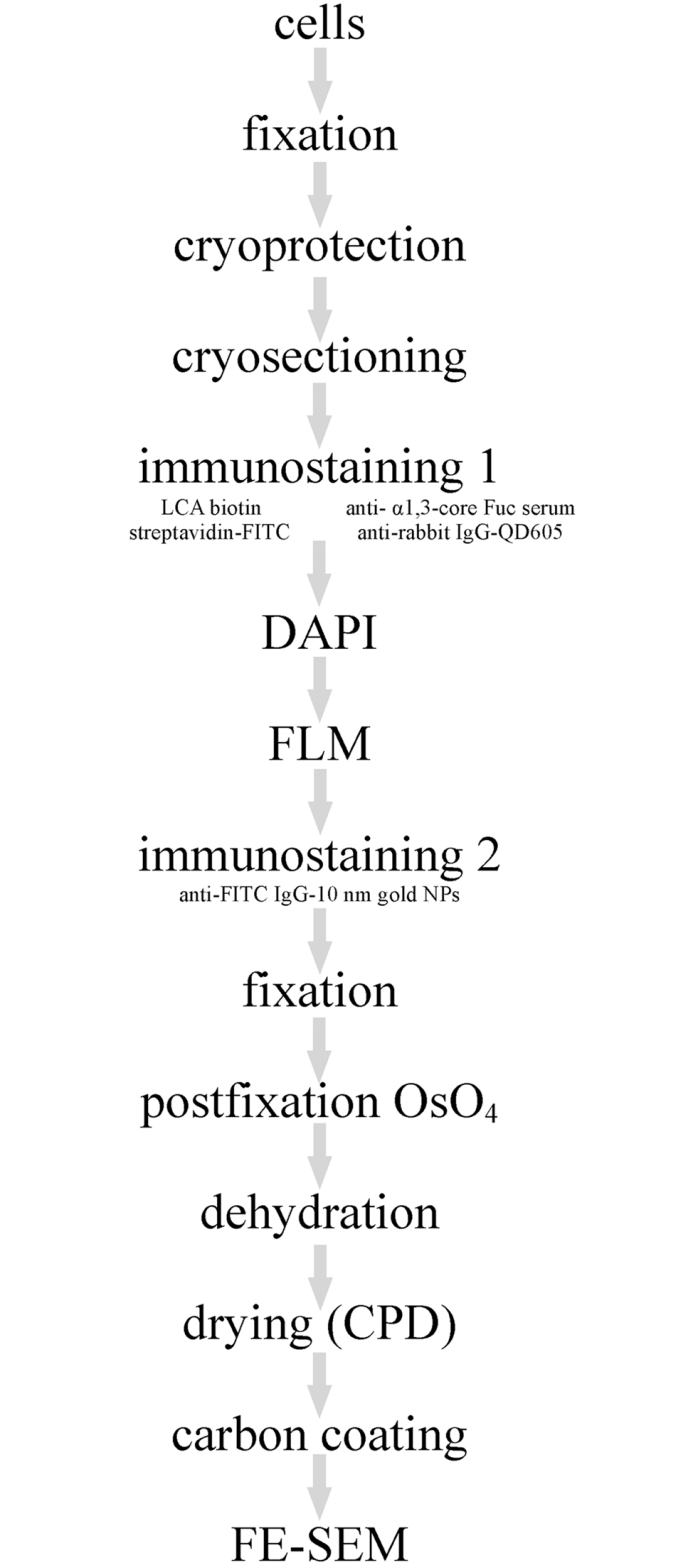
Correlative light and electron microscopy on ultrathin cryosections. Schematic diagram of the procedure.

Non-specific sites were blocked with 3% BSA/0.2% glycine in 0.1 M HEPES for 1 h and labelled with mouse anti-FITC IgG conjugated to 10 nm gold conjugate (Aurion) diluted 1 to 60 in 0.5% BSA for 1 hr. Gold-labelled cryosections were rinsed in buffer, fixed in 2.5% glutaraldehyde for 10 min, washed with three changes in HEPES and incubated in 1% osmium tetroxide for 15 min. Optionally, sections were counterstained with either 1% aqueous uranyl acetate for 10 min and rinsed in water or saturated solution of 50% ethanolic uranyl acetate for 5 min. Cryosections were dehydrated though a series of graded acetone (3 min in each step) and dried using the critical point drying method. Glass slides were mounted on aluminium stubs, carbon coated and observed in FE-SEM JEOL 7401F. A summary of the whole labelling approach is given in Fig 1. SEM images were acquired at accelerating voltage of between 1–8 kV, a working distance (WD, distance from the polepiece of the lens to the sample in focus) of 3–15 mm, and typically with 30 pA of beam current. The BSE images (1280x1024 image size) were captured by a below-lens improved Autrata YAG detector. The SE images were recorded using the conventional Everhart-Thornley detector. The images were aligned in Photoshop. Fluorescence images with a resolution higher than 2000 DPI were covered by flipped SEM micrographs, and the level of the transparency was changed to see both image layers. BSE images of QDs were adjusted using the ACC Image Structure and Object Analyser (Sofo).

## Results

### Identification of region of interest

**Fig 2:**
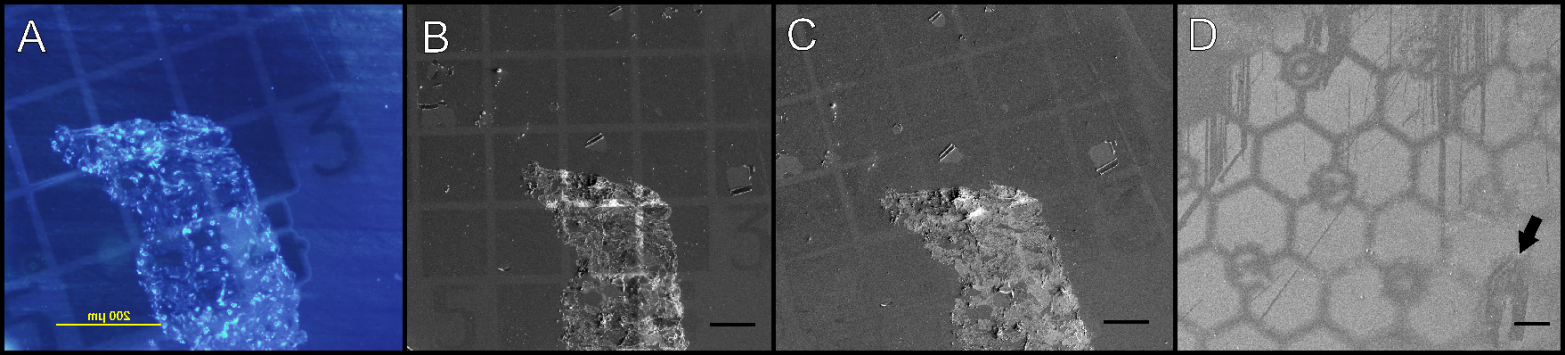
The use of finder grid imprints for the accurate and reliable localization of objects in fluorescence microscopy (A) and SEM (B-D). The imprints were produced on the surface of the glass. In SEM, the carbon imprints were visualized using the SE at 2.8 kV (B) and 1 kV (C). The sections (arrow) on the surface of the gold-based imprints were poorly resolved (D), 1 kV. WD 7.8 (B), 8.6 (C) and 4.7 (D). Bars: 200 μm (A); 100 μm (B-D).

The images were taken together with their position in the indexed carbon pattern that was clearly visible under both the light microscope (Fig 2A) and the SEM (Fig 2B and 2C). In SEM, visualization of the carbon pattern was influenced by the different image contrast between the surface of the glass slide without and with the first carbon layer (for explanation see S1 Fig). This contrast ratio was optimal at an accelerating voltage of 2–5 kV using SE imaging (Fig 2B). However, both edge and charging effects appeared especially in parts of sections placed onto non-conducting areas of the glass slide (parts hidden by grid bars during first carbon coating) (Fig 2B). The SE imaging at 1 kV provided topographical image with reduced charging (Fig 2C). At an accelerating voltage less than 1 kV, the carbon pattern displayed the same brightness level as the clean glass slide. On the other hand, the gold-based imprints on the glass slide led to excellent imaging of the grid pattern but a higher amount of SEs emitted from the substrate, making the localization of the sections almost impossible (Fig 2D, arrow).

**Fig 3:**
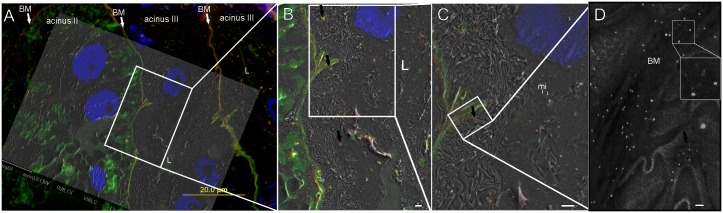
Co-localization of α1,3-core-fucose (red) and *N*-linked α1,6-core-fucose (green) on cryosections of salivary glands isolated from the tick *I*. *ricinus*. Nuclei were stained by DAPI (blue). (A-C) Both glycan structures were present in granules (black arrows) and basement membranes of acini type II and III as shown at different magnification on fluorescent and SE image overlays. Luminal space (L), mitochondria (mi). (D) The BSE image with detail of gold NPs (α1,6-core-fuc) and QDs (α1,3-core-fuc) in inset. FE-SEM at 2.5 kV, 8 mm WD (A); 5 kV and 12 mm WD (B-D). Bars: 1 μm (B, C); 100 nm (D).

In SEM, the carbon imprints and the position of nuclei were used to find the region of interest selected by the fluorescence microscopy. Then, a graded increase in magnification aided substantially in the orientation of observed structures and later for the alignment of fluorescent images and SEM micrographs (Fig 3, S3 Fig).

### Localization of α1,3- and α1,6-linked core-fucose in salivary glands

Both core-α-1, 6-fucosylated N-glycans (detected by LCA, streptavidin conjugated to FITC and 10 nm gold NPs) and core-α-1, 3-fucosylated N-glycans (anti-Fuc IgG and IgG-QDs 605) were co-localized on the basement membranes of the salivary gland acini types II and III, and in the small secretory granules located in the cytoplasm of “F” cells. During tick feeding, this type of cells is extensively transformed and then belongs to the most obvious cell type of acinar type III. “F” cells are responsible for secreting the bulk of the fluid across the salivary glands [19, 20].

**Fig 4:**
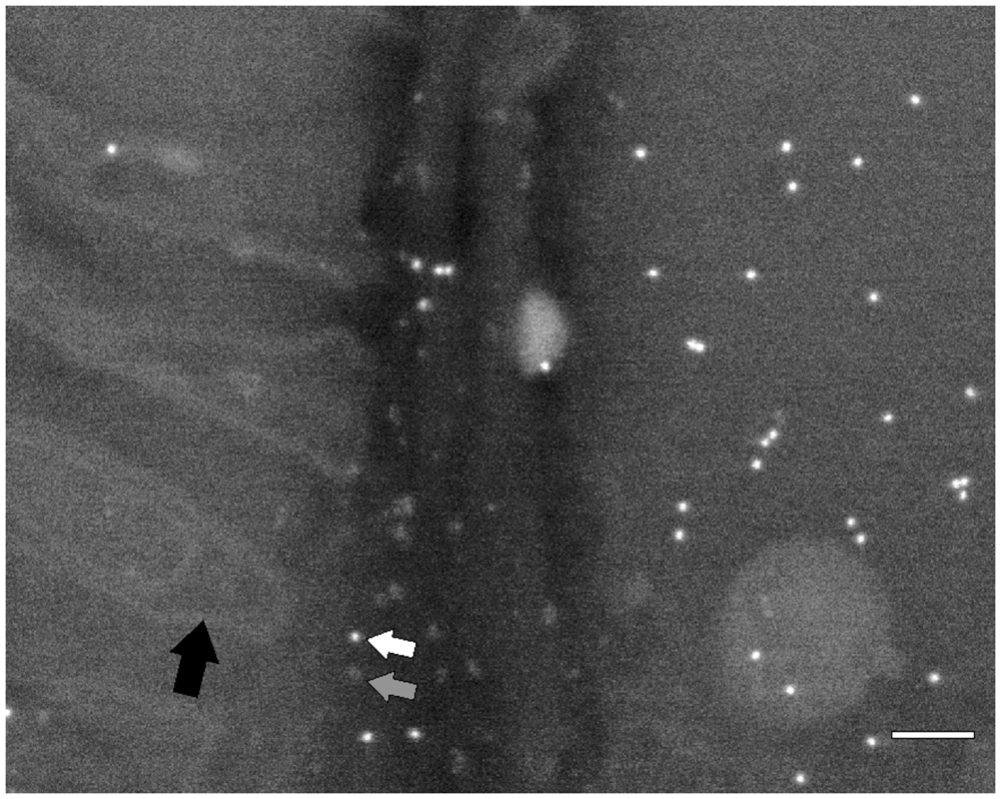
The BSE image of gold NPs (white arrow), QDs (grey arrow) and osmium-stained membranes (black arrow). FE-SEM working at 5 kV and WD of 12–15 mm. Bar: 100 nm.

LCA stained granules were present in secreting cells of the type II acinus (Fig 3A, S3 Fig) whereas α1,3-core-fucosylated structures were here absent. Topographical SE images at low electron energies (1–3 kV) allowed to clearly identify cell organelles such as nucleus, mitochondria, secretory granules (Fig 3B and 3C); whereas ribosomes and other organelles (e.g. endoplasmic reticulum, Golgi compartment) were in most cases indiscernible. The BSE imaging mode at 5 kV and WD of 12–15 mm enable observations of QDs (Figs 3D and 4, grey arrow) and osmium-stained membranes (Fig 4, black arrow). In contrast to QDs, 10 nm gold NPs were imaged with high brightness also by the SE signal; however, particles observed at low electron energies (1–3 kV) were detected only using the conventional SE detector, no signal were found using the upper SE in-lens detector.

**Fig 5:**
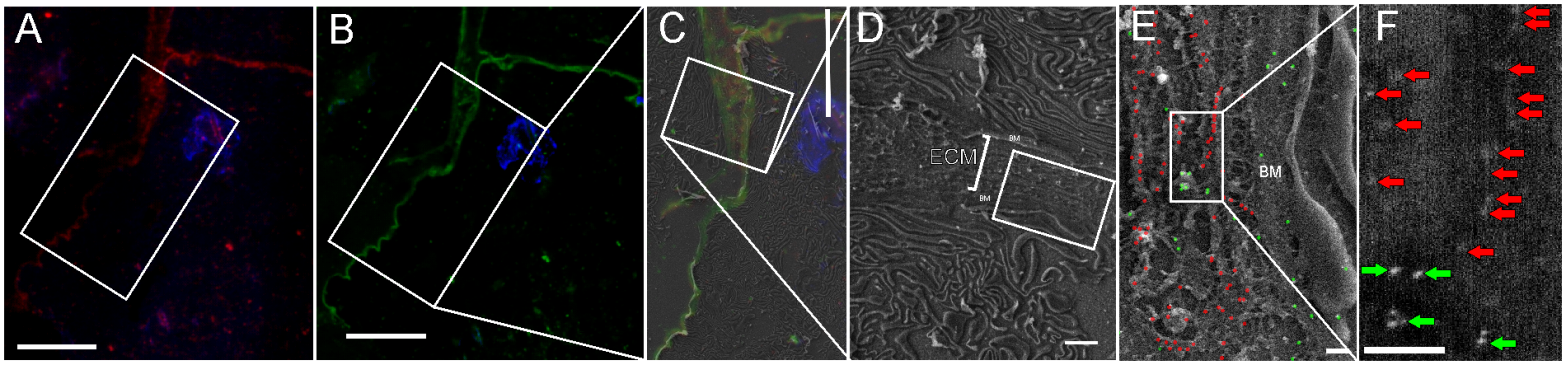
Co-localization of core-fucosylated glycans on cryosections of salivary glands isolated from the tick *I*. *ricinus*. Cryosections were prepared according to the modified Tokuyasu protocol. Fluorescent images show presence of α1,3 core-fucose (A) and α1,6-core- fucose (B). The nuclei were stained by DAPI (blue). (C) The fluorescent and the SE image overlay. (D) The SE image at 2.8 kV reveals presence of two salivary gland acini of the type III surrounded by the basement membranes (BM). The space between acini is filled with extracellular matrix (ECM). (E) The SE image at 4 kV with an overlay showing the position of α1,6 core-fucose (green, gold NPs) and α1,3 core-fucose (red, QD 605) in the extracellular matrix and the basement membrane (BM). (F) Position of gold NPs (green arrows) and QDs (red arrows) was obtained using the BSE mode at 4 kV. Bars 10 μm (A, B, C); 1μm (D); 200 nm (E, F).

Next, core-α-1, 3-fucosylated (red, Fig 5A) and core-α-1, 6-fucosylated N-glycans (green, Fig 5B) were localized in the basement membranes of the acini type II and adjacent extracellular matrix. SE images with typical large depth of field enable clear visualization of extensive basal labyrinth typical for “F” cells (Fig 5C and 5D) and microarchitecture of the extracellular matrix (Fig 5E). The microarchitecture was invisible on cryosections using TEM (S4 Fig). Gold and QD labelled glycoproteins were observed using the BSE mode (Fig 5F).

**Fig 6:**
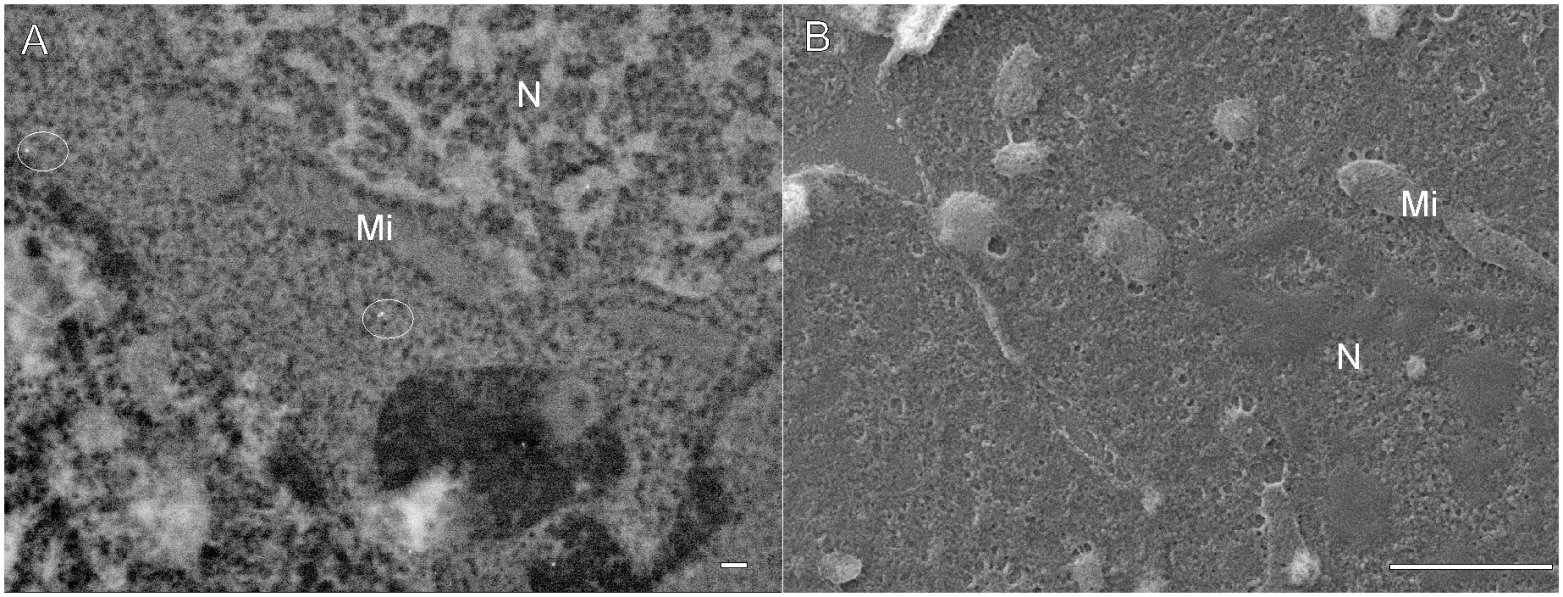
SEM micrographs of cryosections prepared according to a modified Tokuyasu protocol. Sections were post-fixed in OsO_4_, then either unstained (A) or stained in ethanolic uranyl acetate (B), dehydrated, CPD dried and finally carbon coated. (A) BSE, 8 kV, WD 15 mm. (B) SE, 1 kV, WD 8 mm. Nucleus (N), mitochondria (Mi). Bars: 200 nm (A); 1 μm (B).

In order to increase the contrast of biological structures, immuno-labelled sections were stained using either 1% aqueous solution of uranyl acetate or ethanolic uranyl acetate. Even a short incubation of the cryosections on a drop of uranyl acetate substantially increased contrast of the nucleic acids in the BSE mode, but not improved recognition of other organelles. On the other hand, the contrast of the NPs to the surrounding biological structures was decreased (Fig 6A). Topography of unstained (but osmium-postfixed) structures of the similar area imaged in the SE mode is shown in Fig 6B.

## Discussion

In the present study, we showed a mutual distribution of glycan structures modified with α1,6-core-fucose and α1,3-core-fucose in salivary glands of partially fed female *I*. *ricinus*. We showed LCA binding to numerous secretory granules of the acinus type II, whereas α1,3-core-fucosylated glycan structures were found only in several small granules of the type III acinus. Therefore we suggest a minor or no secretion of these immunogenic glycans into a tick host. Both glycan structures were found in the basement membranes surrounding the salivary gland acini and attached extracellular matrix. In *Drosophila*, both α1,6- and α1,3-core fucosylated glycans influence surface interactions of eggs with the sperm *in vitro* [21]. In tick guts, core α1,3-fucose is responsible for the colonization of the tick gut by the pathogenic bacterium *Anaplasma phagocytophilum*, the causing agent of human granulocytic anaplasmosis. In the salivary glands of ticks, this glycan structure was proved by mass spectrometry and fluorescent immunostaining [22].

For purpose of this study, we used alternative fluorescence- SEM approach for observation of labelled Tokuyasu cryosections placed on carbon patterned glass slides. The advantage of using ultrathin cryosections is obvious; they provide labelling with high efficiency and good sharp fluorescent images with a high z-resolution even with conventional wide-field fluorescence microscope. So-called the “Tokuyasu” method involve chemical fixation, infiltration in a sucrose solution and freezing of a specimen in liquid nitrogen. Cryosections are thawed and placed onto a coated grids and finally embedded in a methylcellulose/uranyl acetate thin layer that preserve structures during air drying [23, 24]. Here, the cryosections were post-fixed with osmium tetroxide to increase the contrast of membranes; dehydrated and dried using the CPD method. Regardless, artificial shrinkage/distortion of structures was observed in several cases as revealed during alignments of fluorescent and SEM images. Likewise, sensitivity of structures to electron beam damage could introduce similar artefacts. Recently, ultrathin cryosections were used for co-localization of two fluorescent fusion proteins in both super-resolution fluorescence microscopy and SEM working at BSE mode with accelerating voltage 1.5 kV [25]. Earlier 3-D information was gained using interferometric photoactivated localization microscopy and FIB SEM on 500 or 750 nm tick cryosections [26]. For the FIB-SEM approach, the contrast of cellular structures in cryosections was increased after glutaraldehyde fixation using ferrocyanide-reduced osmium and staining “en block” in lead citrate and uranyl acetate dissolved in polyvinyl alcohol [26]. Here, we found that optimal ratio of NPs signal to background was obtained from a sections post-fixed with 1% osmium tetroxide. If the cryosections were further stained in uranyl acetate, the intense staining led to a low difference in the contrast of NPs (especially QDs) to the cellular structures.

We observed that the carbon-based pattern deposited on a glass surface can be easily detected by means of both fluorescence microscopy and SEM, and it is suitable for rapid and accurate navigation to region of interest in both microscopes. Moreover, the carbon layer prevents charging during SEM imaging. Carbon-based grid pattern on a sapphire disc has been already used for relocation of specific cells in cell cultures after resin embedding [27].

To find the labelled structures in both imaging modalities is the challenge for correlative light and electron microscopy techniques in general. For the precise alignment of fluorescent and SEM images, same objects can be identified based on similar morphological features using the Shuttle & Find tool from Carl Zeiss Microscopy and the MAPS software produced by the FEI Company. Other possibility is the deposition of fiducial markers (e.g. 20 nm gold NPs, fluorescent silica beads) on the surface of samples [28]. Recently, simple, rapid and accurate correlative experiments with metal-patterned glass slides have been described [29]. A new opportunity makes simultaneous light and SEM imaging [12, 30].

We showed that topographic imaging is suitable for accurate interpretation of the ultrastructure and distribution of gold NPs in cryosections. The BSE imaging at 4–5 kV and 12–15 mm working distance enable to see osmium-stained membranes and, in contrast to bright-field TEM, to clearly distinguish QDs (composed from the CdSe core and ZnS shell) located on the cellular structures [31]. It is well known that above 5 kV, the intensity of the BSE signal increases for elements with a high atomic number (such as Au, Pd, Pt, Ag, and Rh) and decreases for elements with a low atomic number [9, 32]. In contrast to QDs, gold NPs of 10 nm in diameter were optimally detected using either the BSE mode (from 4–5 kV and 8–15 mm WD) or the SE imaging from an accelerating voltage of 1 kV (however using conventional Everhart-Thornley detectors of secondary electrons only). However, the BSE mode provided a higher contrast for gold NPs. On the other hand, the use of the low accelerating voltage was beneficial because minimal charging occurred even the sections were only carbon-coated and were placed on the carbon-coated cover slides (see S1 Fig). Both carbon layers provided a sufficient conductivity to minimize the “charging” effect, and therefore, an additional metal coating was not tested. Erlandsen et al. reported that colloidal gold in the range of 6–18 nm in diameter coated with 1 nm of platinum was successfully imaged in the BSE mode; however, charging was not eliminated in the SE (~5 eV) [33]. Similarly, Kopek et al. found that CPD-dried cryosections sputtered with the gold/palladium layer of 1–2 nm reduced the charging [25].

The disadvantages of imaging at a very low accelerating voltage (1 kV) can be worse resolution and small depth of the signal origin (e.g. the SE exit depth is 10 nm for a carbon layer) that may cause NPs of smaller diameters located deeper under the surface of cryosections to be hidden [32]. We confirmed that working distance of the BSE detector was next important parameter. QDs and osmium-stained membranes were imaged in higher contrast with 12–15 mm working distance, whereas morphological details were better seen in this imaging mode with shorter distances.

In conclusion, we described here a novel correlative fluorescence and scanning electron microscopy approach combining modified Tokuyasu cryosectioning method and the use of carbon-patterned glass coverslips. We showed that SEM offers an alternative insights on topography of immunolabelled structures next to widely used transmission EM.

## Supporting Information

S1 Fig Carbon-patterned coverslips. Steps of preparation shown schematically (A). SE image of the carbon pattern at 1 kV (B).
(PDF)

S2 Fig Specificities of used glycan-binding probes. Dominant glycan structures of porcine thyroglobulin unit B (A-B), HRP type II (C), lactoferrin from human milk (D-E), human α1 acid glycoprotein (F-G). Glycan binding motifs with the highest affinity for binding to *Lens culinaris* agglutinin (in white box), and anti-α1,3 core-Fuc antibody (grey). Cleavage sites of endoglycosidase F3 and *N*-glycosidase F enzymes are marked by arrows. For references see S1 Protocol.
(PDF)

S3 Fig Localization of α1,6-core fucose (green) and α1,3-core fucose (red) on ultrathin cryosections of salivary glands isolated from the tick *I*. *ricinus*. The nuclei were stained by DAPI (blue). Fluorescent and SE image overlays (A, B). The higher magnification of the area in boxes A, B (C-D). Gold NPs present on secretory granules of acinus type II and basement membranes (BM) can be clearly distinguished using both the SE (C) and the BSE imaging modes (D), whereas QDs are visible (arrows) only using the BSE (D). Bars: 1 μm (A, B); 100 nm (D, E).
(TIF)

S4 Fig Labelling of α 1,3-core fucose in ultrathin cryosections of salivary glands isolated from the tick *I*. *ricinus*. Highly labeled structures were present in the basement membranes and extracellular matrix. The section were embedded into MC/UA according to Tokuyasu [24]. TEM JEOL 1010 at 80 kV. Bars 200 nm.
(TIF)

S1 Protocol Control experiments. (DOCX)

S1 Table Observed labelling densities of fucosylated glycoproteins. (PDF)

## Abbreviations

FE-SEM: field-emission SEM
SE: secondary electrons
BSE: backscattered electrons
NPs: nanoparticles
QD: quantum dots
WD: working distance
