# Supplementary figures and images for: Correlative Fluorescence and Scanning Electron Microscopy of Labelled Core Fucosylated Glycans Using Cryosections Mounted on Carbon-Patterned Glass Slides
Source: PLoS One. 2015 Dec 21;10(12):e0145034. doi: 10.1371/journal.pone.0145034 (PMC4699470; doi:10.1371/journal.pone.0145034)

A

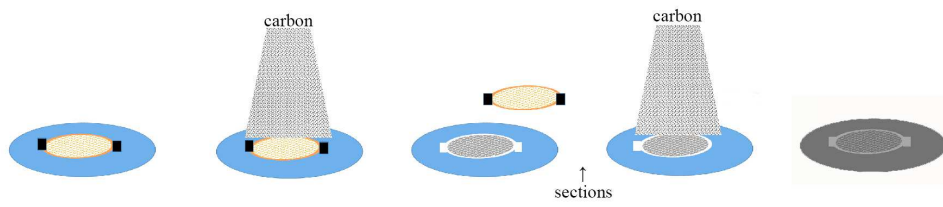

B

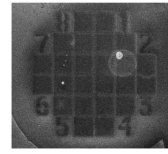

Supplement: S1 Fig — Steps of preparation shown schematically (A). SE image of the carbon pattern at 1 kV (B). (PDF) [file pone.0145034.s001.pdf]

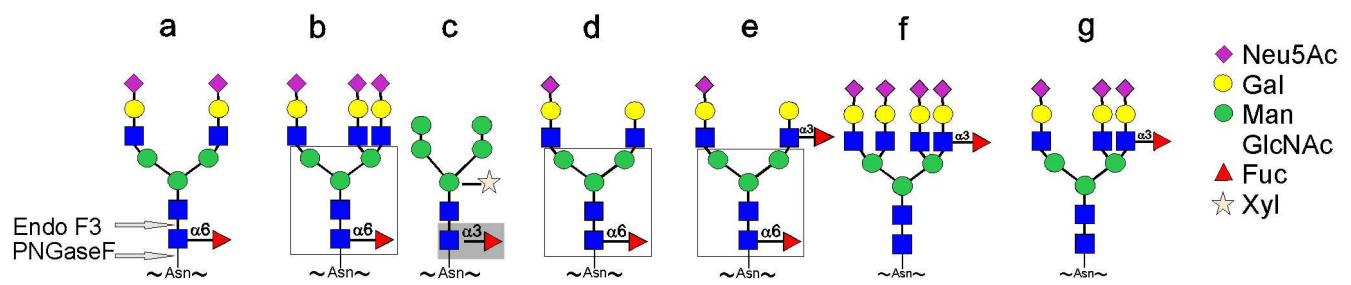

Supplement: S2 Fig — Dominant glycan structures of porcine thyroglobulin unit B (A-B), HRP type II (C), lactoferrin from human milk (D-E), human α1 acid glycoprotein (F-G). Glycan binding motifs with the highest affinity for binding to Lens culinaris agglutinin (in white box), and anti-α1,3 core-Fuc antibody (grey). Cleavage sites of endoglycosidase F3 and N-glycosidase F enzymes are marked by arrows. For references see S1 Protocol. (PDF) [file pone.0145034.s002.pdf]

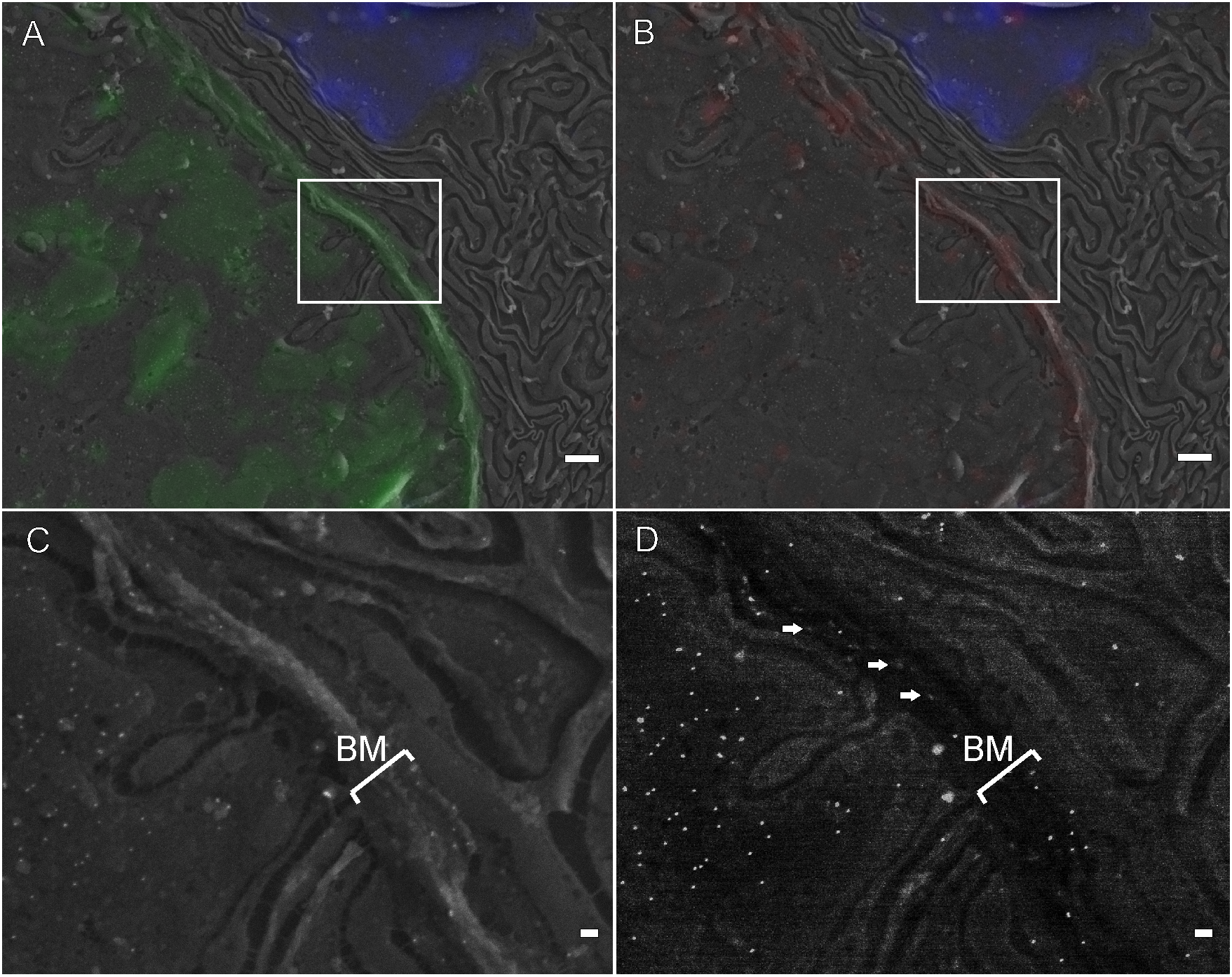

Supplement: S3 Fig — The nuclei were stained by DAPI (blue). Fluorescent and SE image overlays (A, B). The higher magnification of the area in boxes A, B (C-D). Gold NPs present on secretory granules of acinus type II and basement membranes (BM) can be clearly distinguished using both the SE (C) and the BSE imaging modes (D), whereas QDs are visible (arrows) only using the BSE (D). Bars: 1 μm (A, B); 100 nm (D, E). (TIF) [file pone.0145034.s003.tif]

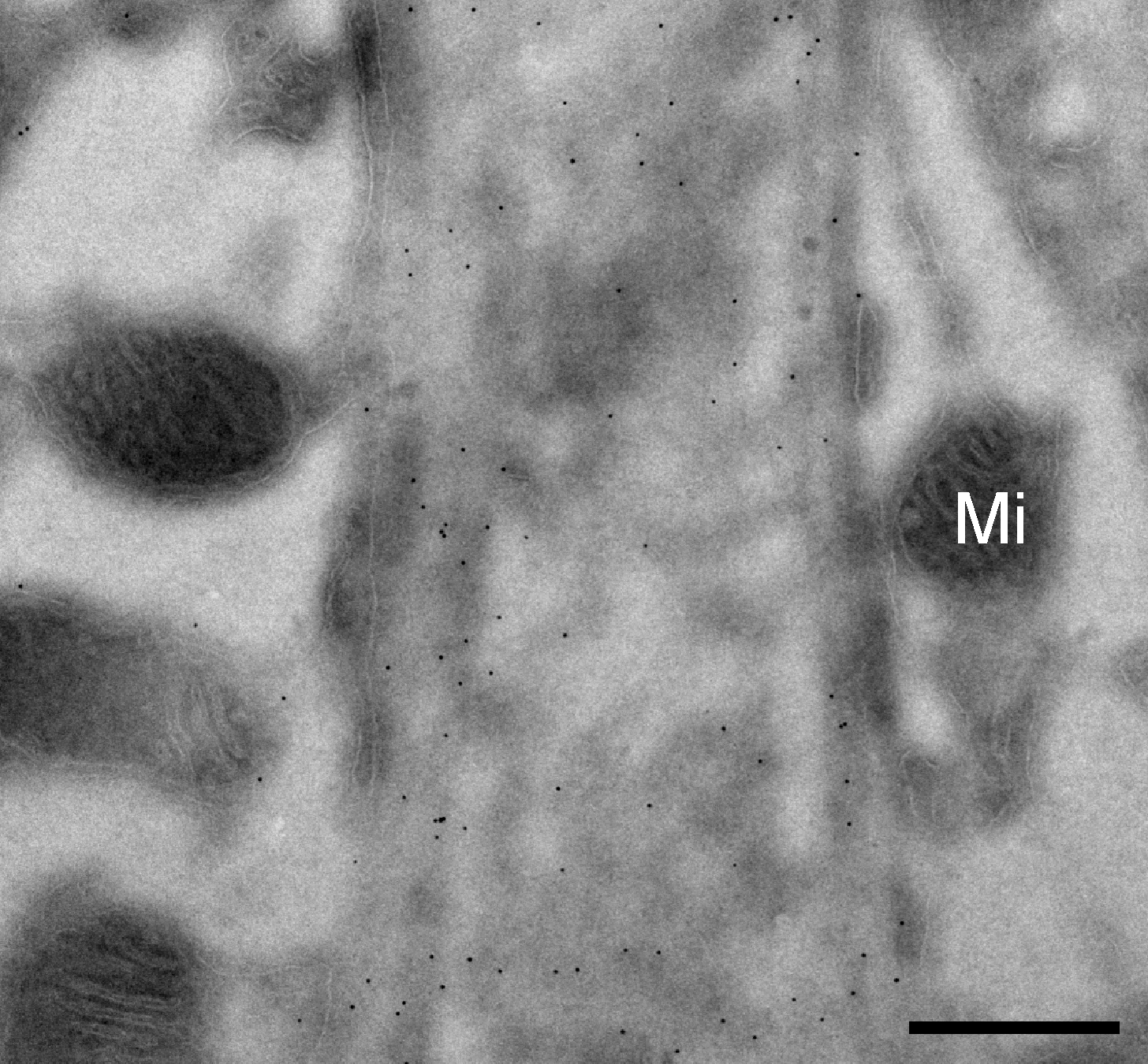

Supplement: S4 Fig — Highly labeled structures were present in the basement membranes and extracellular matrix. The section were embedded into MC/UA according to Tokuyasu [24]. TEM JEOL 1010 at 80 kV. Bars 200 nm. (TIF) [file pone.0145034.s004.TIF]
