# Supplementary material for: Correlative Fluorescence and Scanning Electron Microscopy of Labelled Core Fucosylated Glycans Using Cryosections Mounted on Carbon-Patterned Glass Slides
Source: PLoS One. 2015 Dec 21;10(12):e0145034. doi: 10.1371/journal.pone.0145034 (PMC4699470; doi:10.1371/journal.pone.0145034)
Supplement: S1 Protocol — (DOCX) [file pone.0145034.s005.docx]

**S1 Protocol.**

Firstly, the specificity of glycan labelling was tested on cryosections containing known glycan structures (S2 Figure) [1-4]. Porcine thyroglobulin, human α1 acid glycoprotein, horseradish peroxidase type II and lactoferrin from human milk (all purchased from Sigma-Aldrich) were dissolved separately in 10% gelatine in dH2O at 37°C to obtain 0.17-0.18 M thyroglobulin, 0.55 M horseradish peroxidase, 1.2 M human α1 acid glycoprotein, and 0.26 M lactoferrin. The solutions were cooled down at 4°C, cut into small pieces, immersed in 2.3 M sucrose for 3 days at 4°C and then frozen by plunging into liquid nitrogen. Cryosections containing fucosylated oligosaccharides were blocked with 3% of BSA with 0.2 % glycine in 0.1 M HEPES, and incubated with either biotinylated LCA (25 µg/ml) or anti-α1,3-linked core-fucose serum (3 µg/mL) as described in the section Material and Methods. The sections were washed with 0.5% BSA and incubated for 1 h either in a streptavidin-10 nm gold conjugate (1 to 40 dilution, Aurion) or a protein A-10 nm gold conjugate (1 to 40 dilution, Aurion). Labelled cryosections were rinsed in buffer, water and finally embedded in 1.8% methyl cellulose/0.3% uranyl acetate [5].

Secondly, cryosections of thyroglobulin were enzymatically pre-treated in either 1-2 U of recombinant *N*-glycosidase F (Roche Applied Science) in 0.1 M sodium phosphate (pH 7.5) or endoglycosidase F3 (5 mU, Sigma-Aldrich) diluted in 0.15 M sodium acetate, pH 4.5. Enzymatic reactions were performed for at least 16 hours at 37°C in a moist chamber. Incubation in the corresponding buffer without the enzyme was performed under the same conditions. The sections were then rinsed 6 times with 1% BSA/ 0.05% Tween 20 and labelled by glycan-specific probes as described above.

The results were observed in a TEM JEOL 1010 at 80 kV accelerating voltage and statistically evaluated as follows. Gold NPs were counted on photographs randomly taken at the same magnification using the software program ImageJ (http://rsbweb.nih.gov/ij/). The labelling density values were calculated as the sum of all particles divided by the sum of all areas and expressed as Au/µm^2^. Standard deviations from different photographs were calculated to see the variability in gold distribution among different experiments. Results are summarized in the S1 Table.

References:

1. Nakano M, Kakehi K, Tsai MH, Lee YC. Detailed structural features of glycan chains derived from alpha1-acid glycoproteins of several different animals: the presence of hypersialylated, O-acetylated sialic acids but not disialyl residues. Glycobiol. 2004;14: 431–441.
2. Wuhrer M, Balog CI, Koeleman CA, Deelder AM, Hokke CH. New features of site-specific horseradish peroxidase (HRP) glycosylation uncovered by nano-LC-MS with repeated ion-isolation/fragmentation cycles. Biochim Biophys Acta. 2005;1723: 229–239.
3. Yamamoto K, Tsuji T, Irimura T, Osawa T. The structure of carbohydrate unit B of porcine thyroglobulin. Biochem J. 1981; 195: 701–713.
4. Yu T, Guo C, Wang J, Hao P, Sui S, Chen X, et al. Comprehensive characterization of the site-specific N-glycosylation of wild-type and recombinant human lactoferrin expressed in the milk of transgenic cloned cattle. Glycobiol. 2011; 21: 206–224.
5. Liou W, Geuze HJ, Slot JW. Improving structural integrity of cryosections for immunogold labeling. Histochem Cell Biol. 1996; 106: 41–58.
