# Supplementary material for: Correlative Fluorescence and Scanning Electron Microscopy of Labelled Core Fucosylated Glycans Using Cryosections Mounted on Carbon-Patterned Glass Slides
Source: PLoS One. 2015 Dec 21;10(12):e0145034. doi: 10.1371/journal.pone.0145034 (PMC4699470; doi:10.1371/journal.pone.0145034)
Supplement: S1 Table — (PDF) [file pone.0145034.s006.pdf]

**Glycoprotein**	LCA	Endo F3 LCA	PNGase F LCA	anti-Fuc IgG
Porcine thyroglobulin	34 ± 9.6	8 ± 1.2	0	0
Human α1 acid glycoprotein	0	ND	ND	0
Horseradish peroxidase	0	ND	ND	261 ± 103
Lactoferrin from human milk	0	ND	0	0

Gold NPs/μm^2

not determined (ND)
